# Temporoparietal encoding of space and time during vestibular-guided orientation

**DOI:** 10.1093/brain/awv370

**Published:** 2015-12-30

**Authors:** Diego Kaski, Shamim Quadir, Yuliya Nigmatullina, Paresh A. Malhotra, Adolfo M. Bronstein, Barry M. Seemungal

**Affiliations:** Division of Brain Sciences, Imperial College London, London W6 8RF, UK

**Keywords:** vestibular perception, spatial orientation, time perception, path-integration, temporo-parietal junction

## Abstract

When we walk in our environment, we readily determine our travelled distance and location using visual cues. In the dark, estimating travelled distance uses a combination of somatosensory and vestibular (i.e. inertial) cues. The observed inability of patients with complete peripheral vestibular failure to update their angular travelled distance during active or passive turns in the dark implies a privileged role for vestibular cues during human angular orientation. As vestibular signals only provide inertial cues of self-motion (e.g. velocity, °/s), the brain must convert motion information to distance information (a process called ‘path integration’) to maintain our spatial orientation during self-motion in the dark. It is unknown, however, what brain areas are involved in converting vestibular-motion signals to those that enable such vestibular-spatial orientation. Hence, using voxel-based lesion–symptom mapping techniques, we explored the effect of acute right hemisphere lesions in 18 patients on perceived angular position, velocity and motion duration during whole-body angular rotations in the dark. First, compared to healthy controls’ spatial orientation performance, we found that of the 18 acute stroke patients tested, only the four patients with damage to the temporoparietal junction showed impaired spatial orientation performance for leftward (contralesional) compared to rightward (ipsilesional) rotations. Second, only patients with temporoparietal junction damage showed a congruent underestimation in both their travelled distance (perceived as shorter) and motion duration (perceived as briefer) for leftward compared to rightward rotations. All 18 lesion patients tested showed normal self-motion perception. These data suggest that the cerebral cortical regions mediating vestibular-motion (‘am I moving?’) and vestibular-spatial perception (‘where am I?’) are distinct. Furthermore, the congruent contralesional deficit in time (motion duration) and position perception, seen only in temporoparietal junction patients, may reflect a common neural substrate in the temporoparietal junction that mediates the encoding of motion duration and travelled distance during vestibular-guided navigation. Alternatively, the deficits in timing and spatial orientation with temporoparietal junction lesions could be functionally linked, implying that the temporoparietal junction may act as a cortical temporal integrator, combining estimates of self-motion velocity over time to derive an estimate of travelled distance. This intriguing possibility predicts that timing abnormalities could lead to spatial disorientation.

## Teaser

The cardinal features of vestibular dysfunction are illusory self-motion (vertigo) and spatial disorientation. Testing 18 acute focal cortical lesion patients, Kaski *et al.* show that temporoparietal junction lesions impair vestibular-guided spatial orientation but not self-motion perception. Distinct cortical substrates thus mediate the vestibular percepts of spatial orientation and self-motion.

## Graphical abstract

**Figure:**
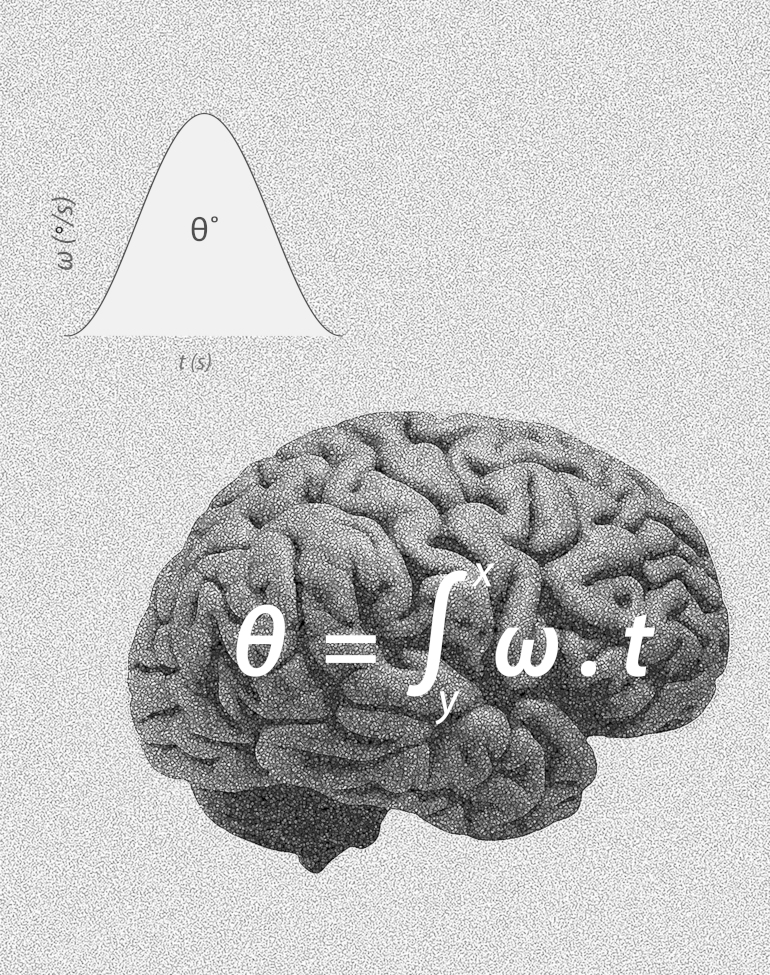
The cardinal features of vestibular dysfunction are illusory self-motion (vertigo) and spatial disorientation. Testing 18 acute focal cortical lesion patients, Kaski *et al.* show that temporoparietal junction lesions impair vestibular-guided spatial orientation but not self-motion perception. Distinct cortical substrates thus mediate the vestibular percepts of spatial orientation and self-motion.

## Introduction

The perception of self-motion lies at the heart of everyday human life. In the light visual cues dominate self-motion perception whereas vestibular cues are critically important when moving in the dark (Glasauer *et al.*, 2002). Higher vestibular functioning can be divided into processes engaged in the perception of self-motion (‘am I moving?’ or ‘how fast am I moving’; vestibular motion perception) and those that mediate orientation in space (‘where am I?’; vestibular spatial perception) (Seemungal, 2014). Both types of vestibular perception can be calibrated by visual input (Aoki *et al.*, 1998) such that when we move in the dark, prior visual calibration will affect our experience of both speed of motion (vestibular motion perception) and distance travelled (vestibular spatial perception).

During head or whole body turns our head motion is detected by the vestibular apparatus that sends a signal of head angular velocity via the vestibular nerve to the brainstem (Fernandez and Goldberg, 1971; Buttner and Waespe, 1981). Signals of head velocity also pass to the cerebral cortex, which mediates the perception of self-motion (Kahane *et al.*, 2003; Nigmatullina *et al.*, 2015)*.* Indeed, primate recordings of thalamocortical pathways relaying vestibular signals of angular motion to the cerebral cortex have so far only yielded vestibular signals of head velocity (Meng *et al.*, 2007) and not of position, suggesting that vestibular spatial signals used in vestibular orientation may be derived from additional processing in the cerebral cortex.

A variety of cerebral cortical areas have been associated with vestibular motion perception (i.e. illusory self-motion) as demonstrated by percepts of illusory rotational self-motion elicited by direct electrocortical stimulation during awake neurosurgery, including the superior temporal gyrus (Kahane *et al.*, 2003), the angular gyrus (Blanke *et al.*, 2000) and the posterior insular cortex (Mazzola *et al.*, 2014). Despite the difficulty in localizing a main vestibular region mediating angular self-motion perception with direct electrical stimulation (potentially reflecting the propagation of electrical activity across brain regions), non-invasive studies in humans, primarily via functional MRI and PET, have suggested a main vestibular cortical region focused in the human homologue of the monkey parieto-insular cortex (Brandt and Dieterich, 1999). Consistent with this notion are primate single neuron data supporting the parieto-insular vestibular cortex as the main cortical region processing vestibular motion signals (Grusser *et al.*, 1990; Chen *et al.*, 2011).

Much less studied is how the vestibular motion signal is transformed to derive vestibular spatial perception required for spatial orientation (‘where am I?’). Primate studies have identified vestibular-position signals in the posterior parietal cortex (Snyder *et al.*, 1998; Klam and Graf, 2003; Snyder and Chatterjee, 2004); however, its relevance for vestibular spatial perception is unclear.

Thus, to identify the neural substrates of vestibular perception (and their underlying mechanisms), we assessed performance in a series of simple vestibular reorientation tasks in the dark in 18 patients with acute hemisphere brain lesions and an age-matched group. We used three different tasks to assess vestibular-spatial perception, vestibular-motion perception, and motion duration perception. We therefore evaluated: (i) whether focal cortical lesions influence self-motion perception and/or vestibular-guided spatial orientation; and (ii) whether the brain regions that mediate the vestibular percepts of self-motion versus spatial-orientation are distinct or overlapping. We used a voxel-based lesion–symptom mapping (VLSM) analysis to determine the relationship between lesion location and performance on the behavioural tasks. Identification of these areas would be important for understanding the neuro-anatomical basis of the vestibular symptoms of vertigo (vestibular-motion perception) and spatial disorientation (vestibular-spatial perception).

## Materials and methods

### Patient demographics, clinical testing and neuroimaging

We tested 18 patients with focal right hemispheric cortical strokes between 3 and 12 days after stroke (Table 1). Data were obtained in the acute phase to limit the effect of brain plasticity obscuring deficits, which thus avoided a heterogenous group of acute and chronic lesion patients. Patients underwent a full neurological and neuro-otological examination [including a head impulse test (Halmagyi and Curthoys, 1988) and thorough oculomotor assessment], and testing for spatial neglect [including star cancellation, copying of drawings (Wilson *et al.*, 1987), and line bisection (18-cm lines)], immediately before taking part in the experiment. For the star cancellation task, 27 stars were presented on either side of the centre of the page. For the line bisection task, line bisection error was calculated as the deviation (in cm) from the midpoint of an 18 cm horizontal line. Note that patients with left hemisphere damage were not tested as dysphasia could interfere with comprehension of the tasks.

**Table 1:** Patient demographics, lesion location and summary of psychophysical performance

Subject	Handedness^a^	Age (yrs)	Sex	Scan	Aetiology	TTS (days)	LHH	EXT	SC R,L	LBB (cm)	Regression slope		Position bias	Temporal bias	Velocity bias
											R	L			
Stroke															
S1	R	71	F	MRI	Infarct	10	No	Yes	26,27	0.9	0.8	0.19	0.24	0.87	0.98
S2	R	60	M	MRI	Infarct	4	No	No	27,27	−0.1	0.9	0.78	0.87	0.62	1.20
S3	R	70	M	MRI	Infarct	3	Yes	Yes	5,0	10.0	0.87	0.78	0.9	0.50	1.00
S4	R	50	F	MRI	Infarct	5	No	No	27,27	−0.2	0.74	0.7	0.96	0.54	1.35
S5	R	48	M	MRI	Infarct	4	No	No	27,20	2.4	0.87	0.21	0.37	0.82	0.89
S6	R	72	F	MRI	Infarct	12	Yes	No	26,27	2.5	1	1	1.00	0.51	1.67
S7	R	68	M	MRI	Infarct	7	No	No	27,27	0.5	0.79	0.84	1.06	0.44	1.16
S8	R	71	F	MRI	Haemorrhage	3	No	No	26,26	−0.8	0.76	0.7	0.92	0.46	0.60
S9	R	65	M	MRI	Infarct	5	No	No	27,27	0.1	0.84	0.81	0.96	0.49	1.58
S10	R	53	F	MRI	Infarct	6	No	No	26,26	−1.0	0.85	0.89	1.05	0.51	0.87
S11	R	80	M	CT	Infarct	3	Yes	No	26,16	6.8	0.68	0.78	1.15	0.49	0.77
S12	L	48	M	MRI	Infarct	4	No	No	27,25	1.2	0.79	0.89	1.13	0.45	1.66
S13	R	68	M	CT	Infarct	5	No	No	26,12	8.4	0.69	0.63	0.99	0.50	1.00
S14	R	71	F	MRI	Infarct	3	No	No	27,27	0.2	0.74	0.35	0.47	0.78	1.07
S15	R	72	F	MRI	Infarct	8	Yes	Yes	21,7	11.7	0.52	0.38	0.73	0.53	1.27
S16	R	52	F	MRI	Infarct	6	No	No	19,19	−0.3	0.67	0.55	0.82	0.54	1.03
S17	R	48	M	MRI	Infarct	6	Yes	No	27,27	0.04	0.99	1.0	0.99	0.42	1.02
S18	R	79	M	MRI	Infarct	5	Yes	Yes	25,15	9.2	0.62	0.54	0.87	0.44	1.15
Control															
C1	R	61	F	N/A	N/A	N/A	No	No	N/A	N/A	0.87	0.78	0.90	0.47	1.18
C2	R	66	M	N/A	N/A	N/A	No	No	N/A	N/A	1.01	0.78	0.77	0.40	0.82
C3	R	55	M	N/A	N/A	N/A	No	No	N/A	N/A	0.73	0.86	1.18	0.50	0.62
C4	R	62	F	N/A	N/A	N/A	No	No	N/A	N/A	0.89	0.84	0.94	0.50	0.75
C5	R	72	M	N/A	N/A	N/A	No	No	N/A	N/A	0.87	0.92	1.06	0.48	1.00
C6	R	54	M	N/A	N/A	N/A	No	No	N/A	N/A	0.92	0.87	0.96	0.49	1.42
C7	R	60	F	N/A	N/A	N/A	No	No	N/A	N/A	0.78	0.84	10.8	0.52	0.79
C8	R	62	F	N/A	N/A	N/A	No	No	N/A	N/A	0.95	0.86	0.91	0.50	0.96
C9	R	68	M	N/A	N/A	N/A	No	No	N/A	N/A	0.97	0.84	0.87	0.42	1.31
C10	L	65	M	N/A	N/A	N/A	No	No	N/A	N/A	0.89	0.93	1.04	0.50	1.18
C11	R	64	F	N/A	N/A	N/A	No	No	N/A	N/A	0.86	0.96	1.12	0.45	1.18
C12	R	66	M	N/A	N/A	N/A	No	No	N/A	N/A	0.9	0.73	0.81	0.52	0.70
C13	R	60	F	N/A	N/A	N/A	No	No	N/A	N/A	0.87	0.85	0.97	0.50	0.66
C14	R	67	F	N/A	N/A	N/A	No	No	N/A	N/A	1.0	0.89	0.89	0.51	1.42
Avestibular															
AV1	R	45	M	N/A	N/A	N/A	No	No	N/A	N/A	0.21	0.02	N/A^c^	0.40	N/A^b^
AV2	R	73	F	N/A	N/A	N/A	No	No	N/A	N/A	0.16	0.32	N/A^c^	0.51	N/A^b^

Data for neglect battery and calculated Position, Time and Velocity biases for stroke patients, controls and avestibular patients.

TTS = time to stroke (days); LHH = left homonymous hemianopia; EXT = extinction; SC = star cancellation. The numbers represent the number of stars cancelled to the right and left (R, L) of the centre of the page (maximum of 27 stars per side); LBB = line bisection bias; Avestibular = complete bilateral peripheral vestibular failure; Regression slope = regression of stimulus versus response angle for Position experiment; Position bias = left/right regression slope; Temporal bias = the probability of the subject indicating that rightward rotations were longer than leftward when the durations of the leftward and rightward rotations were equal; Velocity bias = the ratio between perceptual velocity thresholds for rightward versus leftward chair rotations.

^a^Handedness data collected from patients’ records.

^b^Not applicable as AV1 and AV2 did not perceive the maximum acceleration reached in the Motion task.

^c^As the Spatial task performance regressions for the avestibular patients did not reach significance, it was not appropriate to a provide a ‘position bias’ for these two patients.

All clinical and experimental testing was conducted within a 24-h epoch to minimize the possibility of spontaneous recovery between testing sessions. Stroke patients were on anti-platelet, anti-coagulation, anti-hypertensive and cholesterol lowering drugs, but none were administered acute psychoactive medication. Fourteen age-matched controls with no history of neurological or peripheral vestibular disease were also tested. Throughout the behavioural testing, fatigue was avoided in the patients by careful monitoring and allowing short breaks when necessary.

Two patients with known idiopathic bilateral peripheral vestibular failure confirmed with laboratory testing (bilaterally impaired head impulse tests and absent vestibular ocular reflex responses to bithermal caloric testing and 90°/s velocity steps in the dark) were recruited from our neuro-otology clinics (45-year-old male and 73-year-old female). Both patients with peripheral vestibular failure had no other neurological impairment including no history of cerebrovascular disease. These two patients performed the behavioural tasks to confirm the dependence of these tasks upon vestibular functioning. Written informed consent was obtained from all participants for all experimental procedures as approved by the local research ethics committee.

Brain lesions in stroke patients were imaged by MRI or CT (Patients S11 and S13) and plotted using MRIcro software (http://www.cabiatl.com/mricro/mricro/index.html) on a graphics tablet (WACOM). A T_1_-weighted template consisting of 12 axial slices was used to demarcate the lesions for all patients. Lesion overlap and subtraction were performed in MRIcro. Lesion subtraction was performed by directly comparing those patients who fell outside the 95% confidence intervals (CI) of healthy controls with those who did not, with the latter comprising negative values. This method allows for direct comparison of two groups of patients with one acting as a control, but therefore treats the deficit as a binary phenomenon (Rorden and Karnath, 2004). VLSM was performed with the Non-Parametric Mapping (NPM) software available with MRIcron (http://www.mccauslandcenter.sc.edu/mricro/npm/), and examined all the stroke patients as a single group, treating position bias as a continuous measure. A *t*-statistic was generated for each voxel (Bates *et al.*, 2003) and permutation testing was used to control for family-wise error, as it is thought to be less conservative and potentially more accurate than cluster thresholding in lesion–behavior mapping (Kimberg *et al.*, 2007; Rorden *et al.*, 2009). Permutation testing creates thousands of permutations of the participants’ behavioural scores, and for each permutation the most statistically significant voxel in the brain is recorded, and then rank-ordered (Kimberg *et al.*, 2007).

### Testing vestibular-spatial perception: the Position task

**Figure 1:**
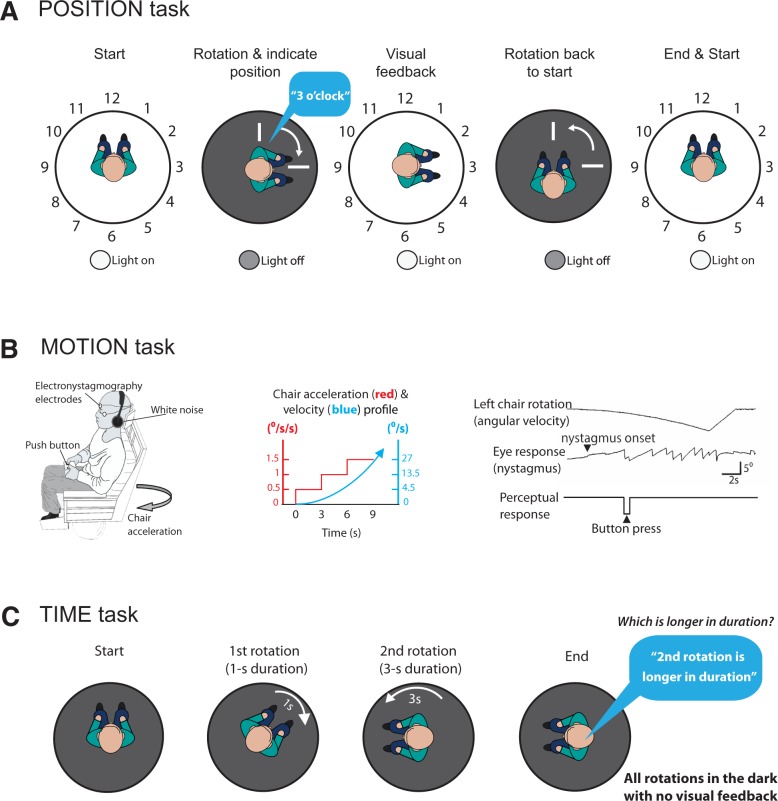
**Experimental protocols and methods.** (**A**) Position task methods. Participants sat in a motorized rotating chair surrounded by a curtain with the numbers of the clock facing the participant. The chair rotated from the 12 o’clock position (‘start’) to another location in the dark, and participants then verbally indicated their perceived clock face position (‘rotation and indicate position’). The lights were then switched on to provide visual feedback (‘feedback’). The lights were then turned off and the chair rotated back to the start position (‘rotation back to start’), and the lights switched on (‘end and start’). (**B**) Motion task methods. Participants were asked to indicate when they perceived motion using button presses (*right*) to indicate right or left as soon as they felt they were moving. Simultaneous ocular motor responses were measured at nystagmus onset, and recorded using electro-oculography. (**C**) Time comparison task methods. Participants were given two distinct angular rotations of varying durations, and asked to indicate which of the two rotations (first or second) was longer in duration.

**Figure 2:**
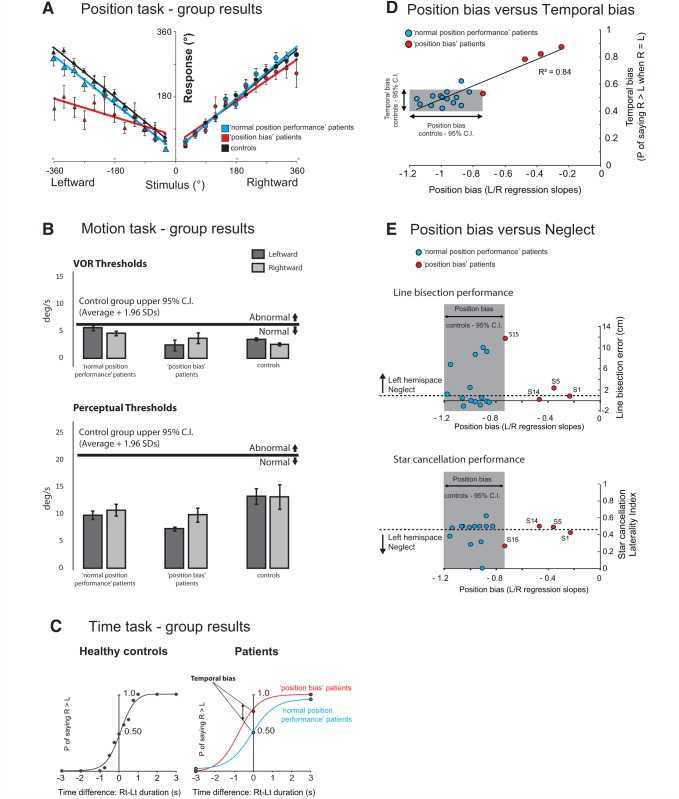
**Behavioural results.** (**A**) Position task results. Grouped response–stimulus position performances are shown for the four patients with a spatial deficit (Patients S1, S5, S14 and S15) (red; ‘position bias’ patients), patients with normal spatial performance (blue; ‘normal position performance’ patients) and age-matched controls (black). Position bias was calculated for each patient from the patient’s response–stimulus position performance regressions, by dividing the leftward regression slope by the rightward regression slope. Vertical bars represent standard errors of the mean. (**B**) Motion task results. Angular velocity thresholds (**°**/s) for ‘position bias’ stroke patients, and ‘normal position performance’ stroke patients, for leftward and rightward rotations. Vertical bars represent standard errors of the mean. The thick horizontal black line delineates the upper limit (group average + 1.96 SD) of the healthy control group motion perceptual threshold. (**C**) Time comparison task results. The temporal bias was obtained by calculating the probability of saying that the rightward rotation was of longer duration than the leftward for healthy controls (black), ‘position bias’ stroke patients (red) and ‘normal position performance’ stroke patients (blue)**.** (**D**) Correlation between bias in the Position task performance and temporal bias in the Time comparison task across all stroke patients (red circles = ‘position bias’ stroke patients; blue circles = ‘normal position performance’ stroke patients). The coefficient of determination (r^2^) relates to all the data points. The shaded region shows the control group’s 95% CIs (95% CIs = average ± 1.96 SD) for position bias (*x*-axis) and temporal bias (*y*-axis). (**E**) The relationship between position bias and neglect. The normal control group’s 95% CIs for a position bias is shaded and four patients (red) have position biases outside of this normal range. For star cancellation performance, laterality index was calculated by dividing the total number of stars observed in the left hemispace by the total number of stars found. Values below 0.46 signify the presence of left neglect (http://www.strokengine.ca/assess/sct).

This task evaluated subjects’ perceived spatial orientation following a rotation in the dark. Subjects were seated in a motorized rotating chair and surrounded by a black curtain suspended from a fixed drum above the chair (Fig. 1A). Numbers from 1 to 12 (angular size 14.8°) were attached to the inside of the curtain, equally spaced by 30°, as in a clock face. The start position was with the subject facing 12 o’clock. White noise was provided via headphones to mask auditory cues and the subjects then rotated in the dark to directly face a number on the curtain. While still in the dark they were asked to say what number they believed they were facing (e.g. ‘3 o’clock’). Visual feedback was then provided by briefly turning the lights on. The lights were switched off and the subject rotated back to the start (12 o’clock). The lights were briefly switched on to reorient subjects to the start position (12 o’clock). Subjects were rotated to the left or the right, through angles of 30° (range 30–360°) in randomized order via raised cosine angular velocities of peak 80°/s, 100°/s or 120°/s. Seventy-two trials were performed in total. Prior to the formal experiment all subjects performed 10 ‘practice’ trials. The spatial performance for each subject was assessed separately for rightward and leftward rotations by plotting a linear regression between stimulus angle (°) versus response angle (°) as shown in Fig. 2. The slopes for the rightward and leftward regressions would thus be approximately equal for patients showing a symmetrical spatial orientation performance. To obtain a measure of symmetry in spatial orientation for each patient, we obtained the ratio of stimulus-response regression slopes for leftward to rightward directions. We called this ratio of leftward regression slope/rightward regression slope the ‘position bias’.

### Testing vestibular-motion perception: the Motion task

This threshold task assessed the ability of subjects to perceive self-motion during whole-body rotations in the dark. This task requires both an intact peripheral vestibular system [the angular velocity (°/s) at which vestibular nystagmus was first seen (criteria as for Seemungal *et al.*, 2004) constituted the vestibular ocular reflex threshold] and the ability to perceive this vestibular signal. Note that when moving in the dark, the vestibular system directly measures our self-motion (head velocity; Fernandez and Goldberg, 1971), but the brain must ‘calculate’ our spatial orientation from velocity signals. Hence, theoretically a deficit in vestibular-motion perception (‘Motion’ task) could cause a deficit in vestibular-spatial perception (‘Position’ task).

Using a modified version of the technique described (Seemungal *et al.*, 2004; Cutfield *et al.*, 2011), subjects were exposed to angular rotations of increasing acceleration (0.5°/s^2^ every 3 s) from a stationary start, either to the right or to the left. They were asked to depress one of two buttons (right or left) as soon as they perceived the movement and its direction (Fig. 1B). The time taken to press a button (perceptual response), and the time to onset of nystagmus (vestibular ocular response) were recorded. Normal values for the vestibular-ocular reflex and perceptual thresholds were determined from the 14 age-matched healthy controls that took part in this study and reported as the 95% CIs [mean threshold + 1.96 standard deviations (SD); Fig. 2B]. Velocity bias in the Motion task was calculated as a ratio of perceptual velocity thresholds (chair velocity at time of button press to indicate perceived self-motion) for rightward versus leftward chair rotations (i.e. velocity bias = velocity thresholds for leftward / rightward rotations).

### Testing motion duration perception: the Time comparison task

This task assessed subjects’ ability to discriminate the duration of self-motion. Given that updating our spatial orientation is vestibular-dependent when we turn in the dark, we assessed whether this updating of perceived position from velocity cues involved estimates of motion duration. Subjects were seated in a motorized rotating chair in total darkness, with white noise played through headphones (Fig. 1C). Subjects were specifically asked to concentrate on the duration of self-rotations in the dark. In addition, the numbers on the visual surround were removed prior to this task so that subjects did not engage any visual-spatial representations during the task. In the task, subjects were given two distinct rotations of varying duration, and asked to indicate which of the two rotations was the longer in duration, first or second (Fig. 1C). For healthy controls, peak angular velocities of 60°/s and 90°/s were used, with durations of either 1, 1.5, 1.75, 2, 2.5, 3 or 4 s to produce relative time differences between rotations of 0, 0.5, 0.75, 1, 2, or 3 s. Rotation pairs were randomly assigned to ensure equal numbers of starts to the left or to the right. To ensure that all patients were able to perform the task without fatiguing, in the patient group we only tested time differences of 0 (e.g. a 2-s rotation to the right versus a 2-s rotation to the left) and 3 s (e.g. a 4-s rotation to the left versus a 1-s rotation to the right). For the patient group the rotations were of amplitude 0–180° and peak angular velocities of 60°/s or 90°/s. For equal rightward and leftward rotation durations, an unbiased subject’s probability of the indicating that rightward rotations were longer than leftward (*P*_Right>Left_) should be 0.5. Subjects’ temporal bias was thus calculated as the probability of the subject indicating that rightward rotations were longer than leftward when the durations of leftward and rightward rotations were equal (termed *P*_Right>Left_).

### Data recording and analysis

All signals were recorded at a sampling rate of 250 Hz for off-line analysis. Eye movements were recorded during the motion perception task using DC-coupled horizontal electro-oculography (EOG) with EOG signals filtered at 30 Hz. Eye movement traces showed no evidence of spontaneous nystagmus in the light or dark, and no vestibular oculomotor asymmetries were detectable in any patient. A chair tachometer, which was used to record chair velocity for all tasks and angular chair displacement, was read from an off-axis angular encoder whose signal was recorded with an accuracy of <1° for rightward and leftward rotations. Specifically each degree of angular rotation was represented by four computer units during digital sampling of the chair position signal. Psychometric probability curves were plotted to display the time perception data using Sigmaplot (Systat, version 11).

Parametric statistics including *t*-test, one-way ANOVA and repeated measures ANOVA were used to compare between group responses in the experiments.

## Results

### Position task

The group ‘position biases’ (for patients, and controls) are shown in Fig. 2A and the position bias for each patient is shown in Table 1. Table 1 shows that 4 of 18 stroke patients (Patients S1, S5, S14 and S15) manifest a position bias beyond the healthy controls’ 95% CI performance range. Figure 2A shows the group performance for the four patients with spatial deficit (‘position bias’ patients) on the Position task, 14 patients with no deficit, and 14 controls. Of note, the number of position estimate errors in later trials were no different to those of earlier trials suggesting that fatigue did not affect performance throughout the 72 trials.

**Figure 3:**
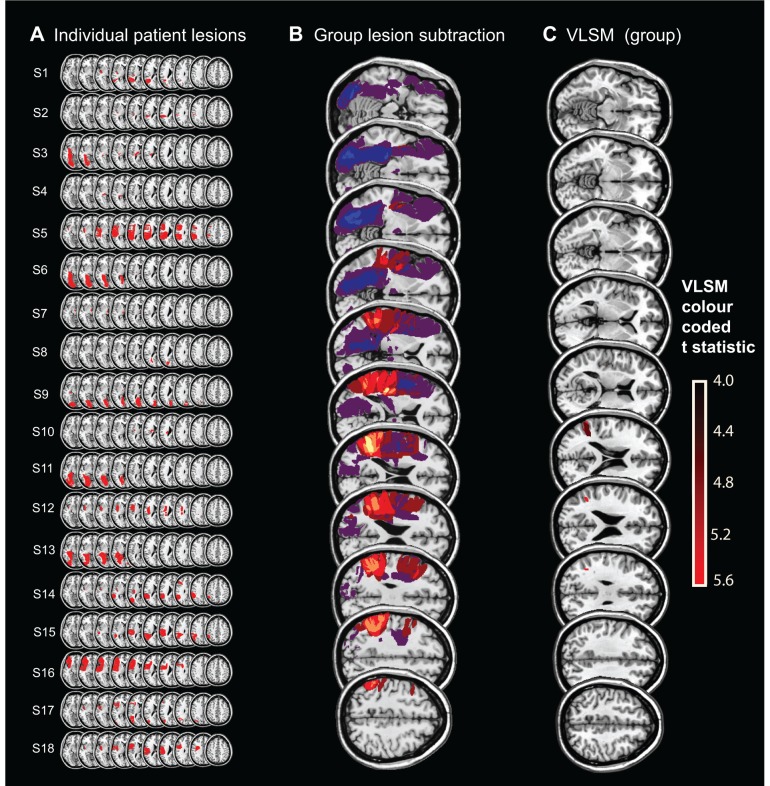
**Brain lesion maps and analysis.** (**A**) Lesion map of all stroke patients (Patients S1–S18). (**B**) Lesion subtraction analysis for Patients S1–18 localized the Position task deficit to the temporoparietal junction (TPJ) shown in yellow. (**C**) VLSM analysis. For the VLSM, a *t*-test was performed at each voxel (using 1000 permutations and a *P*-value of 0.05) only in voxels that were damaged in at least three individuals. The bar on the far right gives the colour coding for the significance level for the VLSM analysis (units = *t*-values and only voxels with *t* > 4 are displayed). The most significant regions were in the angular gyrus (MNI: 38, −53, 30 to 43, −53, 24; *t* = 5.16) and just reaching the superior temporal gyrus (60, −53, 20), with further less significant voxels in the middle temporal gyrus (48, −51, 20; *t* = 4.62).

The patients’ lesion distributions are shown in Fig. 3A. Lesion subtraction analysis (Rorden and Karnath, 2004) contrasted the four stroke patients (Patients S1, S5, S14 and S15) with position bias (outside 95% CIs of healthy control data; positive values) with the 14 patients who showed no position bias (negative values) when compared with healthy controls. This demonstrated that position bias was only observed in patients whose lesion included the temporoparietal junction (TPJ) and that patients without damage to this region (Fig. 3B, yellow) did not manifest a deficit on the position bias task.

We performed a VLSM analysis, including all 18 stroke patients and examined position bias as a continuous measure throughout the whole group. This analysis showed that the most significant regions (Fig. 3C, red) associated with a spatial deficit (‘position bias’) were the angular gyrus [Montreal Neurological Institute (MNI): 38, −53, 30 to 43, −53, 24; t = 5.16] and just reaching the superior temporal gyrus (60, −53, 20), with further less significant voxels in the middle temporal gyrus (48, −51, 20; t = 4.62). The 14 patients without TPJ-overlapping lesions had normal Position task performance.

### Motion task

Patients S1–S18 showed normal ocular motor (vestibular-ocular reflex) and motion perception thresholds (Motion threshold task, Fig. 2B) as compared to age-matched healthy controls (one-way ANOVA with factors Group and Response, *P* > 0.1). In addition the patients’ motion perceptual thresholds were within the normal values previously reported in the literature (Cousins *et al.*, 2013). In particular, the average perceptual thresholds for leftward and rightward rotations for the four position bias patients (Fig. 2B) were within the 95% CI for the controls’ responses and not statistically significant (unpaired, two-tailed *t*-test, *P* > 0.05). Given the evidence suggesting that the insula is involved in processing vestibular signals of head motion, we also compared motion perceptual thresholds for contralesional and ipsilesional rotations between eight insular and 10 non-insular lesion patients and 14 healthy controls. However, we found no difference in motion perception between these groups [one-way ANOVA for six conditions; three groups × two rotation directions; *F*(1,5) = 1.048, *P* = 0.40].

### Time comparison task

An unbiased subject’s probability of indicating that rightward rotations were longer than leftward when the durations of leftward and rightward rotations were equal (*P*_Right>Left_) should be 0.5. For the control healthy aged-match group, the average *P*_Right>Left_ was 0.48, which was significantly different from the spatial-deficit patient group’s average *P*_Right>Left_ of 0.76 (*P* < 0.0001, unpaired *t*-test; Bonferroni corrected significance level of *P* < 0.016; Fig. 2C). Three of the spatial deficit patients (Patients S1, S5 and S14) had temporal biases >8 SD larger than the control group average. Figure 2D displays the position bias and temporal bias results for the spatial deficit and non-deficit patients, showing that patients’ position bias and temporal bias are correlated. Notably, the comparison of motion durations between rotations of the same direction (right versus right, and left versus left) was uniform across all subjects, with preserved detection of 3-s differences between rotations. Finally, the order of presentation did not bias subjects’ responses for equal duration rotations (one-way ANOVA with factors Group and Response; *P* = 0.58).

### Spatial neglect and Position task performance

Clinical data for the neglect testing are shown in Table 1 (star cancellation and line bisection bias). The occurrence of neglect is clearly dissociated from the spatial deficit (Fig. 2E). Interestingly, two patients with TPJ lesions (Patients S1 and S5) manifested symptomatic topographical disorientation at the time of their stroke, the former having been found wandering on the ground floor of the hospital unable to find her way back to the ward on her third day of admission, and the latter complaining that he was unable to find his way back to the bed from the ward toilet. Patient S1 (who had the most severe position bias deficit) underwent additional testing for representational neglect immediately following her participation in the experiment. When instructing the patient to describe landmarks on the road where she lived using Google Street View (https://www.google.com/maps/views/streetview), she reliably described all landmarks on the left and right sides of the street when describing it from opposite viewpoints, ruling out any significant degree of representational neglect.

## Discussion

Our data show that: (i) deficits in vestibular-guided spatial orientation (the Position task) were manifest only in those patients whose lesions involved the TPJ; (ii) patients with spatial disorientation on the Position task showed a temporal bias (in the Time comparison task) congruent to the direction and magnitude of their spatial disorientation; (iii) vestibular-sensed self-motion perception was not affected by focal brain lesions; and (iv) vestibular-guided spatial orientation deficits were not secondary to spatial neglect. Put simply, patients whose lesion involved the TPJ underestimated both their travelled distance and motion duration during vestibular-guided leftward turns in the dark when compared to rightward rotations.

### Linking spatial and motion duration data

We consider two main possibilities to explain the apparent relationship between deficits in time comparison and spatial orientation in patients with damage to the TPJ.

#### A non-mechanistic link

First, it may be that the mechanisms mediating the perception of motion duration (Time task) and spatial orientation (Position task) are not functionally linked but that their apparent linkage (shown in our results) could arise simply because their neural correlates occupy an overlapping brain location in the TPJ. As such, that Time task deficits manifest in TPJ lesion patients would reflect a general timing role of the TPJ rather than any specific role in spatial orientation. Supporting its general role in timing, the TPJ has been linked with estimating and perceiving duration (Davis *et al.*, 2009) as well as temporal comparisons as required for the Time task (Battelli *et al.*, 2003; Woo *et al.*, 2009; Cappelletti *et al.*, 2011).

A non-mechanistic hypothesis makes two additional general predictions: (i) timing deficits, without spatial deficits, could be isolated in at least some patients; however, we did not observe isolated deficits in either spatial orientation or temporal estimation; and (ii) common deficits in time and spatial performance could arise as a result of deficits in cognitive processes that could simultaneously influence spatial and/or temporal perceptual performance, e.g. attention mechanisms and the mental number line deviation (Zorzi *et al.*, 2002; Corbetta and Shulman, 2011; Karnath and Rorden, 2011). Neglect is a disorder of attention and results in an inability to report, respond or orient to novel or meaningful stimuli presented on the contralesional side (Mort *et al.*, 2003). Against the proposition that the TPJ may mediate the binding of spatial and temporal information via attentional mechanisms (Snyder and Chatterjee, 2004), we found no correlation between neglect measures and either Position or Time task performance (Fig. 2E). Apart from our data, others have also shown that neglect can dissociate from navigational deficits (Philbeck *et al.*, 2001). A mental number line deviation could potentially explain the bias observed in the position task in the TPJ lesion patients, as this task (unlike the Motion and Time conditions) directly involved processing of numerical information. However, individuals with number biases secondary to focal lesions manifest numerical bias, but not increased variability, and this is incongruent with the observed bias and increased variability in the TPJ lesion patients’ performance on the Position task. Moreover, Aiello *et al.* (2012) recently showed, using a clock representation, that ‘defective processing of smaller magnitudes in a number interval was present both when these magnitudes were mapped on the left and the right side of a mental visual image’. Their data predict that in our Position task, patients showing hypometric responses for leftward rotations should also display hypermetric responses for rightward rotations, a prediction that is not supported by our findings (Fig. 2A). Taken together, cognitive phenomenon such as neglect and the mental number line distortion cannot explain our results.

#### A mechanistic link

Alternatively, a mechanistic hypothesis dictates that the neural processes underlying motion duration perception and spatial orientation are functionally linked, predicting a tight relationship between vestibular spatial perception and motion duration perception, with congruent deficits in both. In line with this, we found that patients with worse spatial performance showed a worse temporal performance, suggesting a tight overlapping in neural correlates between these two functions. A link between spatial and temporal estimates may be of particular relevance for spatial orientation and ‘path integration’—the process by which the distance travelled within the environment is derived from motion cues (visual, somatosensory, and vestibular) (Mittelstaedt, 1980; McNaughton *et al.*, 1996). The concept of a mathematical integration is particularly relevant for spatial orientation under vestibular guidance in the dark because theoretically, by continuously sampling our self-motion velocity and summing this velocity information over time (i.e. an integration of velocity over time), the brain could derive an estimate of our travelled distance. It follows that the brain, and specifically the TPJ, may encode vestibular-guided movement in a form that preserves the relationship between travelled distance (s), velocity of motion (v) and duration of motion (t), i.e. s = ∫v.dt (Berthoz *et al.*, 1995; Seemungal *et al.*, 2007). While lesions of the dominant angular gyrus lead to impairments of explicit mathematical calculation (e.g. Gerstmann’s syndrome), our data may suggest that the non-dominant angular gyrus plays a role in implicit mathematical calculation such as the derivation of position from velocity and time. The concept of a ‘cortical integrator’ draws parallels to the well-established brainstem integrator for eye movement control (Pastor *et al.*, 1994). An impaired cortical integrator could thus underlie certain types of egocentric topographical disorientation syndromes associated with focal posterior right hemisphere lesions (Aguirre and D’Esposito, 1999).

The use of timing estimates to derive a spatial estimate is suggestive of an internal model (Green *et al.*, 2005). Indovina *et al.* (2005) provided evidence for the use of an internal model for vestibular perception (the detection of gravitational motion kinematics), specifically involving the TPJ. Bosco *et al.* (2008) also found that perturbing TPJ function using transcranial magnetic stimulation (TMS) impaired subjects’ timing of interception in response to a moving visual target, but only when its motion kinematic profile was consistent with acceleration under gravity. During yaw-axis (horizontal plane) vestibular-guided angular navigation, we previously found that repetitive TMS to left or right posterior parietal cortex disrupted encoding of contralateral angular position and motion duration, but not angular velocity perception (Seemungal *et al.*, 2009).

Any data supporting a mechanistic link between time estimation and spatial orientation can however only be correlative; whether our data linking temporal and spatial estimates with TPJ lesions are an epiphenomenon or are mechanistically linked would require a selective perturbation of time perception. This appears beyond current approaches as, so far, all experimental manipulations of time perception invariably involve or affect other sensorimotor modalities. Nevertheless, our data clearly show that the TPJ mediates human vestibular-guided spatial orientation and motion duration perception.

Behavioural testing of patients with left hemisphere lesions is often complicated by the presence of aphasia, leading to uncertainties in the degree of comprehension of the behavioural tasks, and the communication of responses to a given stimulus. Nevertheless, vestibular navigation performance following transient disruption of left hemisphere functioning using TMS (Seemungal *et al.*, 2008) suggests that a rightward position bias deficit could occur in patients with left hemisphere lesion. On the other hand, given the right hemisphere dominance in the vestibular cortical network (Dieterich *et al.*, 2003; Seemungal *et al.*, 2008), the potential for a rightward position bias with a left hemisphere lesion may be masked by intact right hemisphere function (Sack *et al.*, 2005).

### Motion perception is unaffected by focal cortical lesions

An unexpected finding was that none of the patients we tested showed any abnormality in self-motion perception, including those whose lesions involved the human homologue of the monkey parieto-insular vestibular cortex (see ‘Results’ section), thought to be the main cortical locus involved in processing vestibular signals of head motion (Grusser *et al.*, 1990; Dieterich and Brandt, 1993; Brandt and Dieterich, 1999). A recent large study in acute stroke (Baier *et al.*, 2013) found no effect of focal posterior insular lesions on a vestibular perceptual function (of the subjective visual vertical). Perhaps, tellingly, there have been no prior reports of isolated deficits of vestibular motion perception with focal hemispheric lesions in the human homologue of the monkey parieto-insular vestibular cortex or elsewhere in the brain (although this omission could represent a failure of commissioning the appropriate studies rather than a failure to report negative results). Overall, our data showing a lack of an effect of acute unilateral hemispheric lesions on self-motion perception could suggest that vestibular motion perception is bilaterally encoded in the cerebral cortex (and requiring bilateral lesions to cause a deficit), a notion supported by a recent neuroimaging study in healthy humans (Nigmatullina *et al.*, 2015).

### Summary

Our data show that the TPJ is critically involved in vestibular spatial perception (‘where am I?’), but not vestibular motion perception (‘am I moving?’). It follows that these two faculties are separately encoded in the brain. This predicts that deficits in spatial disorientation arising from cortical disturbances could occur separately from deficits in self-motion perception. Our finding that deficits in vestibular spatial function were congruent with deficits in motion duration perception is intriguing, and leads us to speculate that the TPJ may act as a cortical temporal integrator that combines estimates of self-motion velocity over time to mediate the updating of travelled distance when navigating in the dark and under vestibular guidance.

## Abbreviations

TPJ: temporo-parietal junction
VLSM: voxel lesion symptom mapping
